# Silent Moyamoya disease - A rare case report

**DOI:** 10.1016/j.radcr.2021.03.019

**Published:** 2021-04-09

**Authors:** Juna Musa, Masum Rahman, Ali Guy, Angela Guy, Kristi Saliaj, Abu Bakar Siddik, Fjolla Hyseni, Ketjana Elezi, Ina Kola, Anisa Cobo, Ilir Ahmetgjekaj

**Affiliations:** a Department of Surgery Physiology and Biomedical Engineering, Mayo Clinic, 200 1st St, SW Floor 8 Rochester, MN, 55905, USA; b Department of Neurological Surgery, Mayo Clinic, 200 1st St, SW Floor 8, Rochester, MN, 55905, USA; c Department of Physical Medicine and Rehabilitation, New York University, School of Medicine, NYU Medical Center, 550 1st Avenue, New York, NY, 10016, USA; d Health Emphasis California School of Professional Psychology Alliant International University, 1000 South Fremont Ave, Unit 5, Alhambra, CA, 91803, USA; e Mother Teresa hospital university, Rruga e Dibrës 372, Tirana, AL, 1000, Albania; f Department of Pain Medicine, Mayo Clinic, 4500 San Pablo Rd S, Jacksonville, FL, 32224, USA; g Department of Urology, NYU Langone Health, 11th and 12th Floors, 222 E 41st St, New York, NY, 10017, USA; h Pharmacist and master in Health management, University of Medicine, Tirana, AL, 1000, Albania; i Department of Burns and Plastic Surgery, Mother Teresa Hospital, Rruga e Dibrës 372, Tirana, AL, 1000 Albania; j University of Medicine, Tirana, AL, 1000, Albania; k University Clinical Center, Clinic of Radiology, University For Business and Technology –Radiology, Pristina, Kosovo

**Keywords:** Cerebrovascular arteriopathy, Moyamoya syndrome, Ischemic stroke, Cerebral, Angiography, Radiological findings

## Abstract

Moyamoya is a rare cerebrovascular disorder marked by chronic, gradual blockage of the circle of Willis arteries, leading to characteristic collateral vessels, specifically cerebral angiography. The disease can develop in children and adults, although there are different clinical characteristics. Moyamoya disease occurs mainly in Japanese people but has been reported in all races of varying age distributions and clinical features. As a reason, Moyamoya disease has been under-recognized as a cause of hemorrhagic and ischemic strokes in Western nations. There is no proven solution at this time, and there is debate over current care methods. The authors identify a case of a 40-year-old male with a small subcutaneous nevus-like mass in his left orbit who was diagnosed with Moyamoya disease.

## Introduction

Moyamoya disease (MMD) is a rare, chronic cerebrovascular arteriopathy, characterized by a progressive stenosis and occlusion of the distal segment of the internal carotid artery (ICA) and its branches, the middle cerebral artery (MCA) and/or the proximal anterior cerebral artery (ACA) [1,2]. This progressive stenosis affecting important parts of the cerebral vasculature, leads to the development of an abnormal collateral circulation at the base of the brain [1,2].

The angiographic image of this network of vessels has the appearance of a hazy puff of smoke or moyamoya in Japanese [3]. Moyamoya is a descriptive term, first coined by Suzuki and Takaku in 1969, to describe this particular angiographic picture and to suggest that it accounted for a new pathological entity, that was later named Moyamoya disease [3]. A distinction is usually made between *Moyamoya disease* (the occlusion is bilateral and idiopathic or unilateral) and *Moyamoya syndrome* (the occlusion is associated with an underlying systemic condition) [4]. Diagnostic criteria for Moyamoya disease were revised to encompass both bilateral and unilateral presentations, due to an increasing number of patients presenting with a unilateral occlusion that eventually progressed to a bilateral involvement [2].

The prevalence of MMD is higher in East Asian countries compared to other regions, ranging from 10.5/100,000 in Japan to 16.1/100,000 in South Korea [1]. The incidence has a bimodal distribution pattern with two peaks, one at the age of 10 years old, and the other one later in life, at the ages of 35-50 years old [1,5]. Generally, the incidence in females is slightly higher than in males [1,5].

The pathophysiology of MMD remains to be elucidated, however some susceptibility genes have been identified, RNF213 being one of the most important ones associated with familial MMD [1]. Other implicated genes include BRCC3/MTCP1 and GUCY1A3 genes [5]. An autoimmune association in the pathogenesis of MMD has been hypothesized, due to elevated levels of multiple autoimmune antibodies [1,5]. An overexpression of proangiogenic factors and other cytokines has also been found in MMD cohorts [5]. These findings suggest that they contribute to the development of the collateral circulation, as well as the progression of the disease, further implicating chronic arterial inflammation in the pathogenesis of MMD [1,5].

The clinical presentation of MMD includes transient ischemic attacks (TIA), ischemic stroke, hemorrhagic stroke, epilepsy, headache and cognitive dysfunction [1,2]. Most pediatric patients present with ischemic symptoms including TIA-s and ischemic strokes [1]. Nevertheless, there have been previous reports of asymptomatic cases of MMD, in literature. A well-established definition of asymptomatic MMD has not yet been agreed upon, however most studies define the absence of clinical and imaging findings associated with ischemic or hemorrhagic strokes, in the context of imaging findings suggestive of Moyamoya disease, as asymptomatic MMD [6], [7], [8], [9]. Cognitive impairment affecting intelligence, spatial abilities, verbal working memory and number manipulation is present in asymptomatic patients [8]. Data from several studies shows that asymptomatic MMD is a progressive pathological entity, promoting disturbances in cerebral hemodynamics that eventually lead to ischemic or hemorrhagic strokes [6], [7], [8], [9].

The golden standard for establishing a definitive diagnosis of MMD and evaluating its progression is cerebral angiography [1,^2^,5]. However, owing to the invasive nature of the procedure, magnetic resonance angiography (MRA) remains an excellent diagnostic option. Its findings have been shown to be consistent with those of cerebral angiography and high resolution nuclear MRI can effectively distinguish between atherosclerosis and MMD [1]. Evaluation of cerebral perfusion through single-photon emission computed tomography (SPECT), positron emission tomography scan (PET scan) and arterial spin labeling is an important step to provide a more global assessment of the condition, the prognosis and inform the treatment [5].

There is no definitive treatment for MMD. Prognosis of MMD depends on the severity of the symptoms, the precise location and the extent of the occlusion, as it dictates the clinical presentation and guides the therapeutic efforts. In symptomatic patients, management consists in the improvement of the cerebral perfusion through surgical revascularization. Ischemic and hemorrhagic stroke protocols are applied in acute presentations. Conservative management includes the use of aspirin as prophylaxis for further strokes or thrombotic events, in addition to anticonvulsant and analgesic medications to manage the seizures and headaches [5]. There is no consensus in the right approach to managing asymptomatic MMD. Conservative management of these patients includes lifestyle modifications, particularly concerning stroke-related risk factors, as well as anticoagulation therapy. [6,^7^,9] The use of anticoagulants in asymptomatic patients remains controversial, with some studies supporting its use and others suggesting they may increase the likelihood of hemorrhagic strokes [6,^7^,9]. Regular follow-up with MRI angiography is crucial in this population, as it allows for an early detection of vascular abnormalities and a timely surgical revascularization, thus preventing serious neurological sequelae [6,^7^,9].

## Case presentation

A 40-year old male presented to the ophthalmologist's office regarding a small subcutaneous nevus-like mass in his left orbit. The patient was asymptomatic. Ophthalmic examination was normal, without any findings of proptosis or ocular motility defects. Pupils were equal and reactive to light, bilateral visual acuities were 10/10 with no signs of elevated intraocular pressure. On inspection, a small, palpable, firm, non-tender mass was evident in the anteroinferior aspect of the orbit. The rest of the examination, as well as routine laboratory tests were within normal limits. His past medical history and family history were unremarkable.

Due to concerns about a possible orbital tumor, a MRI of the head was ordered. It showed a small lesion, adjacent to the lacrimal duct that was ultimately confirmed to be a hemangioma. More importantly, the MRI revealed an occlusion of the right middle cerebral artery (MCA) along with the presence of a collateral circulation network, highly suggestive of silent unilateral Moyamoya disease (MMD).

**Fig. 1:**
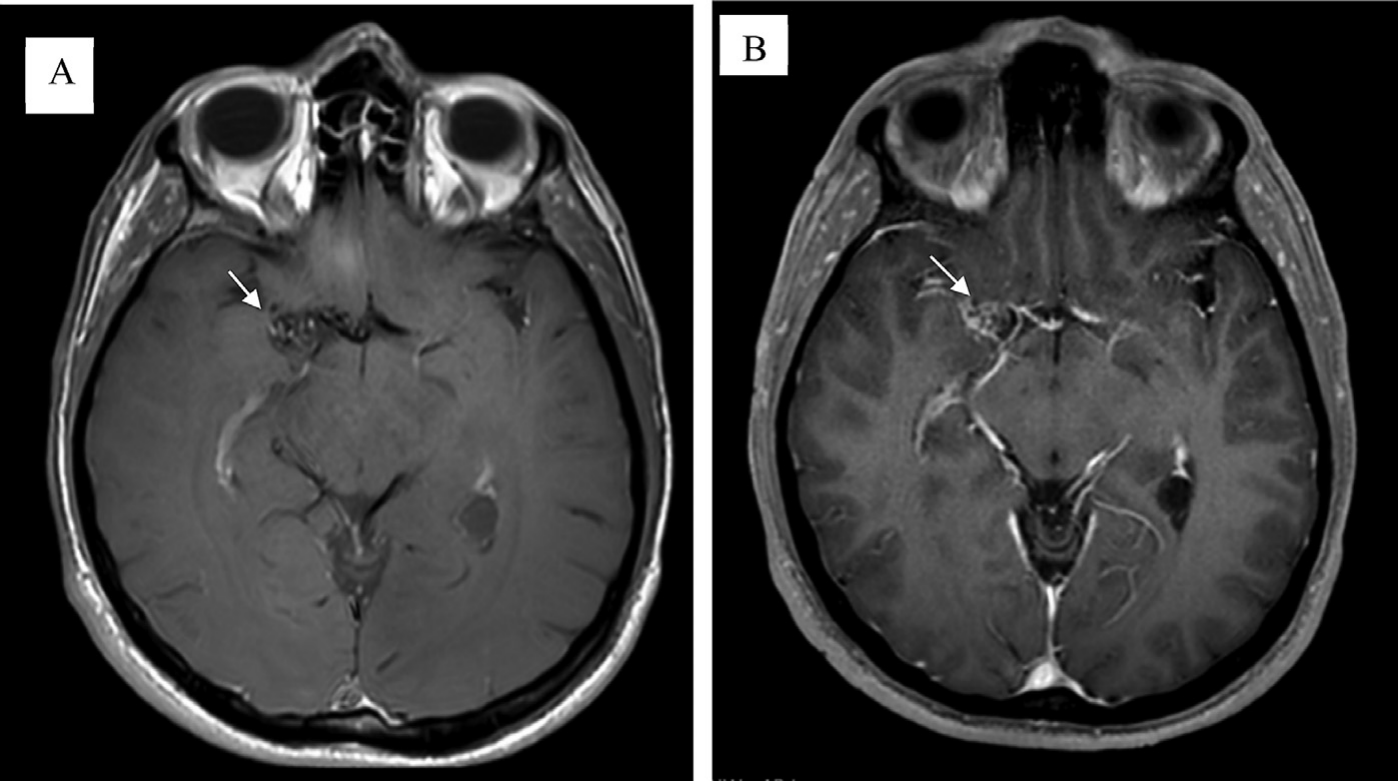
Magnetic resonance (MR) image. MRI Axial T1 post contrast in right MCA shows increased number and size of lenticulostriate vessels “puff of smoke” (images A and B) (white arrows) that represent collateral circulation as a consequence of tapering in right middle cerebral artery.

**Fig. 2:**
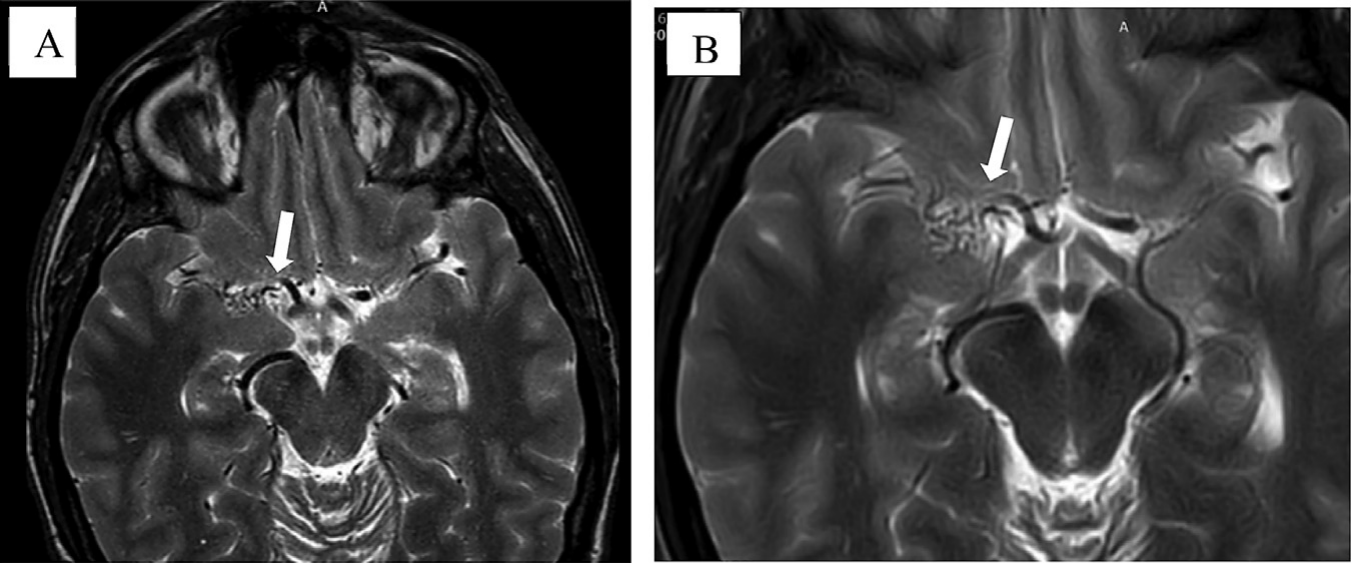
MRI Axial T-2 weighted images show no ischemic lesion. The flow-void signal of the right middle cerebral artery is sluggish, with diffuse net of small vessels denoting collaterals. (images A and B) (white arrows)

**Fig. 3:**
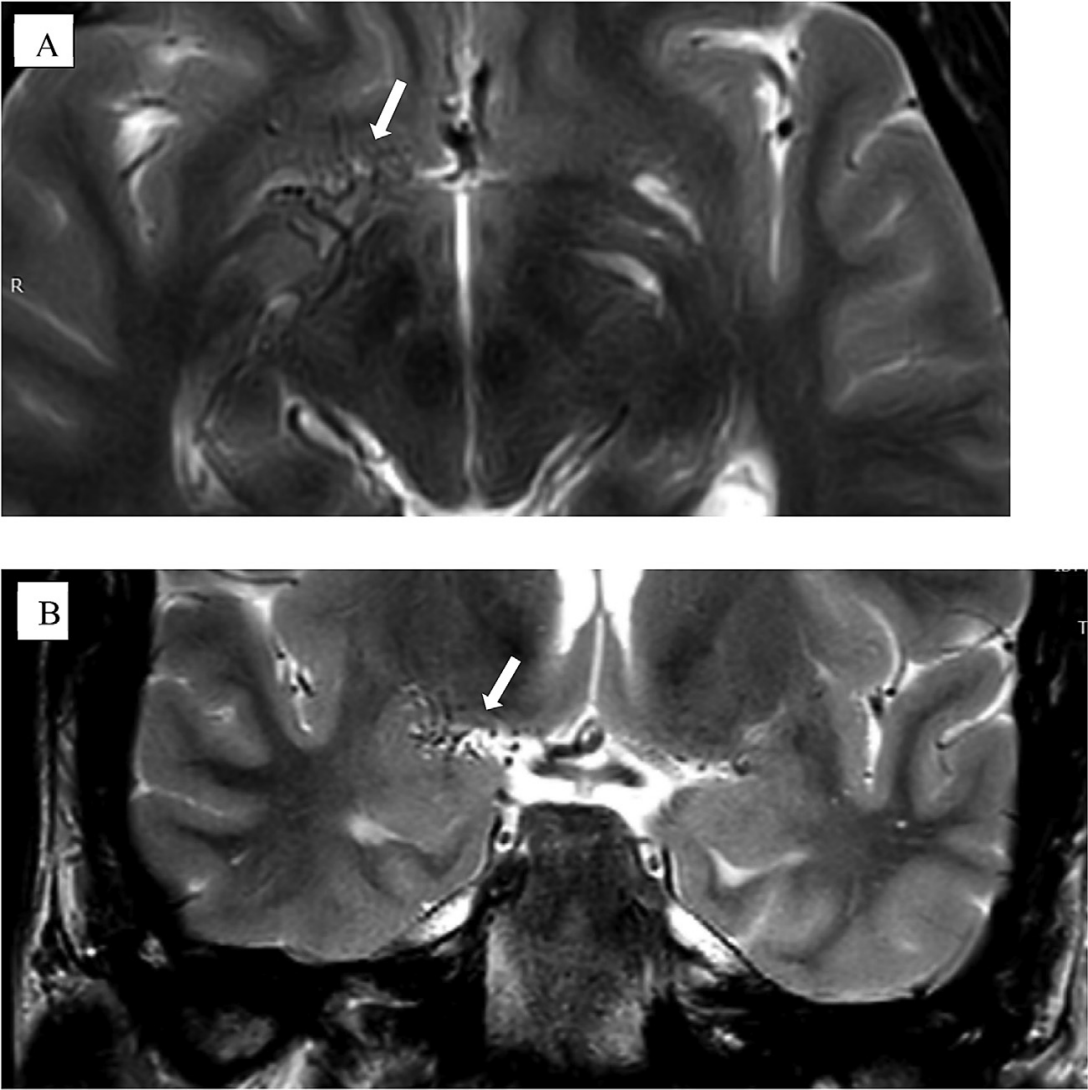
Coronal T2 weighted image (A and B) (white arrows) shows right MCA occlusion with flow voids presenting diffuse net of small vessels denoting collaterals.

Axial T1 and T2-weighted images (Fig. 1) showed a diffuse network of dilated lenticulostriate vessels, the so-called “puff of smoke”. This network represents the collateral circulation, developing in response to the occlusion of the right middle cerebral artery (MCA). The occlusion of the right MCA with flow voids was also present in the axial and coronal T1 and T2-weighted images (Fig. 1-3).

**Fig. 4:**
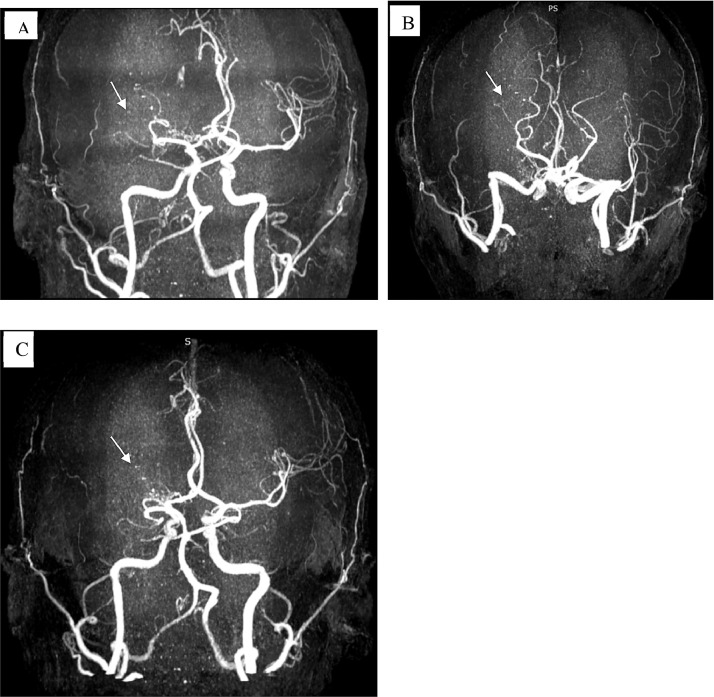
Time-of-flight magnetic resonance angiography (TOF-MRA) images (A, B and C) (white arrows) demonstrating complete obliteration of the right middle cerebral artery.

Time-of-flight magnetic resonance angiography (TOF-MRA) confirmed the complete stenosis of the right MCA (Fig. 4).

The imaging findings were entirely incidental, as the patient was completely asymptomatic and had not experienced any symptoms or signs indicative of Moyamoya disease.

The patient was managed conservatively, with recommendations for lifestyle modifications, as well as stroke-related risk factors. It was decided against anticoagulation therapy, as there was no imaging evidence, at present, of vascular anomalies associated with a potential ischemic event. Biannual MRI and/or MRA examinations were recommended as well.

## Discussion

Moyamoya disease (MMD) represents a unique phenomenon with characteristic angiographic findings in the absence of other systemic illnesses. MMD was first described in Japan in 1957. It predominantly affects East Asians; however, it has been described in populations across the world. Moyamoya disease yields a bimodal age distribution with peaks around five years of age and in the mid-40s, and is almost twice as common in females than males [10].

Patients typically present with acute cerebrovascular events, including ischemic stroke, transient ischemic attacks (TIA), intracranial hemorrhages, occasionally seizures, and cognitive decline [1,^2^,8]. The progressive cerebral hypoperfusion eventually promotes cognitive impairment, intellectual decline or mental retardation [2]. Seizures and persistent headaches are also common manifestations in the pediatric population [1,2]. Half of the adult patients present with hemorrhagic strokes, due to the presence of aneurysm and pseudoaneurysm in the fragile and dilated vessels and the other half with ischemic strokes [1,2]. The anterior circulation is predominantly affected, nevertheless involvement of the posterior circulation has been well-documented and is associated with a poorer prognosis [2]. Hemorrhagic strokes are more common in Asian patients compared to Caucasians ones [2]. Incidental findings of asymptomatic MMD have been reported in several studies [6,^7^,[9], [10], [11], [12]. Patients with angiographic evidence of MMD without any ischemic or hemorrhagic episodes are classified as asymptomatic or silent MMD [6], [7], [8], [9].

One research on asymptomatic MMD has shown that 20% of asymptomatic cases have silent cerebral infarction ipsilateral to the site of Moyamoya vessels. And 40% of cases revealed disturbed cerebral hemodynamics, including moderate to severe reduction of the cerebral perfusion reserve, higher O2 extraction and low performance during acetazolamide challenge throughout 43.7 months follow-up [7]. The same study suggests these anomalies may be independent indicators of future ischemic strokes [7]. Alterations in both cerebral blood flow (CBF) and cerebrovascular reactivity (CVR) to acetazolamide were present in 10% of the asymptomatic population in a nation-wide survey in Japan [6]. Recent studies with susceptibility-weighted MRI have demonstrated that 15-44% of adult patients have silent microhemorrhages in the basal ganglia, thalamus, and periventricular white matter and suggest they may be an independent risk factor for future hemorrhagic strokes [6]. Cognitive impairment involving intelligence, memory, spatial ability and number manipulation is present in asymptomatic patients [8].

Although some patients showed stable disease, MMD is progressive in most instances, with a 13.3% annual stroke rate, and most of the patients experience recurrent strokes. To date, no medical therapy has been proven as prophylactic in MMD. Surgical revascularization has been offered in ischemic MMD to augment cerebral blood flow and prevent future ischemic events. While in hemorrhagic MMD, theoretically, it is believed that bypass procedures prevent the recurrent hemorrhages by lowering the long-term hemodynamic stress on the collateral vasculature [13]. In a study on asymptomatic MMD, 7 of 34 patients suffered an ischemic stroke, TIA, or hemorrhagic stroke, who did not undergo revascularization surgery. Interestingly, in another study shown, none of the patients who were treated with surgical revascularization presented with any MMD symptoms on follow-up [7,14].

At the present, a definitive management approach for asymptomatic MMD hasn't been established, due to limitations in the early diagnosis and insufficient follow-up data from small scale studies. Conservative management of these patients has been proposed by a number of studies [6,^7^,9]. This approach consists in modifications of risk factors including hypertension and smoking, with anticoagulation regimens being supported by some studies, while others undermine their effectiveness [6,^7^,9]. Periodic MRI and/or MRA follow-up is indispensable in this subgroup of MMD [6,^7^,9]. Surgical treatment in asymptomatic cases is only advised in patients with radiological evidence of cerebral hypoperfusion [6,^7^,9]. In patients without imaging findings associated with cerebral perfusion anomalies, it is generally believed that the risks of the intervention outweigh the benefits.

## Conclusion

The incidence and prevalence of MMD are rising with improvements in imaging as well as other diagnostic tools. Molecular analysis of the Moyamoya vessels may improve the understanding of MMD and lead to earlier diagnosis. A proper understanding of patients' natural history with Moyamoya disease and the benefit of the various treatment modalities are needed [14,15].

MMD has become a more established cause of stroke for children and adults. To obtain the best result in patients, it is crucial to identify the disease early in its development through characteristic radiological findings with prompt therapy institutions. Surgery may be useful, mainly if Moyamoya disease is diagnosed at an early stage. Further forward-looking studies are therefore required. Operational revascularization benefits for prospective patients are likely to be improved by enhancements of surgical procedures, perioperative treatment, and anesthesia. In adult patients with Moyamoya disease, careful neurologic and radiologic long-term follow-up is vital to avoid further stroke and improve performance [10,16].

## Patient consent

Patient consent has been obtained.
